# Rare case of ascites several years after liver transplantation

**DOI:** 10.1093/gastro/goac076

**Published:** 2022-12-05

**Authors:** Liesbeth Deroo, Anne Hoorens, Xavier Verhelst, Helena Degroote, Hans Van Vlierberghe, Anja Geerts

**Affiliations:** Department of Gastroenterology and Hepatology, Ghent University Hospital, Ghent, Belgium; Department of Pathology, Ghent University Hospital, Ghent, Belgium; Department of Gastroenterology and Hepatology, Ghent University Hospital, Ghent, Belgium; Department of Gastroenterology and Hepatology, Ghent University Hospital, Ghent, Belgium; Department of Gastroenterology and Hepatology, Ghent University Hospital, Ghent, Belgium; Department of Gastroenterology and Hepatology, Ghent University Hospital, Ghent, Belgium

## Case report

A 67-year-old male patient with a history of liver transplantation presented with complaints of progressive abdominal distention. He had a history of alcoholic cirrhosis complicated by hepatocellular carcinoma for which he underwent orthotopic liver transplantation in 2011. Afterwards, he developed end-stage renal disease, requiring hemodialysis in 2017. In 2018, the diagnosis of high-risk essential thrombocythemia (ET) (presence of the *JAK2(V617F)* mutation and advanced age without a history of thrombosis) was made. In the absence of thrombocytosis and presence of severe renal anemia, a treatment with once-daily aspirin, to prevent thrombosis, was initiated. Despite the chronic kidney disease (CKD) and diabetes, further follow-up of his liver transplantation was favorable. He quit smoking in 2006 and stopped using alcohol in 2010. There was no relevant familial history.

Nine years after transplantation, the patient came on routine consultation with a complaint of abdominal distention for 1 month. There were no other symptoms. Physical examination revealed only a distended but not painful abdomen. There were no signs of edema on his lower limbs. Blood analysis revealed low hemoglobin (7.9 g/dL), normal platelet and leukocyte counts, and a normal C-reactive protein level. Electrolyte levels were in the normal range and kidney function was decreased (creatinine 4.38 mg/dL). Liver enzymes were slightly disturbed (aspartate aminotransferase 20 U/L, alanine aminotransferase 17 U/L, gamma-glutamyl transpeptidase 208 U/L, alkaline phosphatase 124 U/L, lactate dehydrogenase 212 U/L). The international normalized ratio was 1.16. Viral serology test results of human immunodeficiency virus, hepatitis B, hepatitis C, and cytomegalovirus were negative. Ultrasonography of the abdomen showed ascites and splenomegaly, but no signs of cirrhosis. Abdominal veins were patent. Magnetic resonance imaging of the liver showed no signs of cirrhosis. A diagnostic paracentesis (ascitic tap) showed a serum–ascites albumin gradient of 1.8, indicating portal hypertension. There was no evidence of malignant cells.

**Figure 1.**
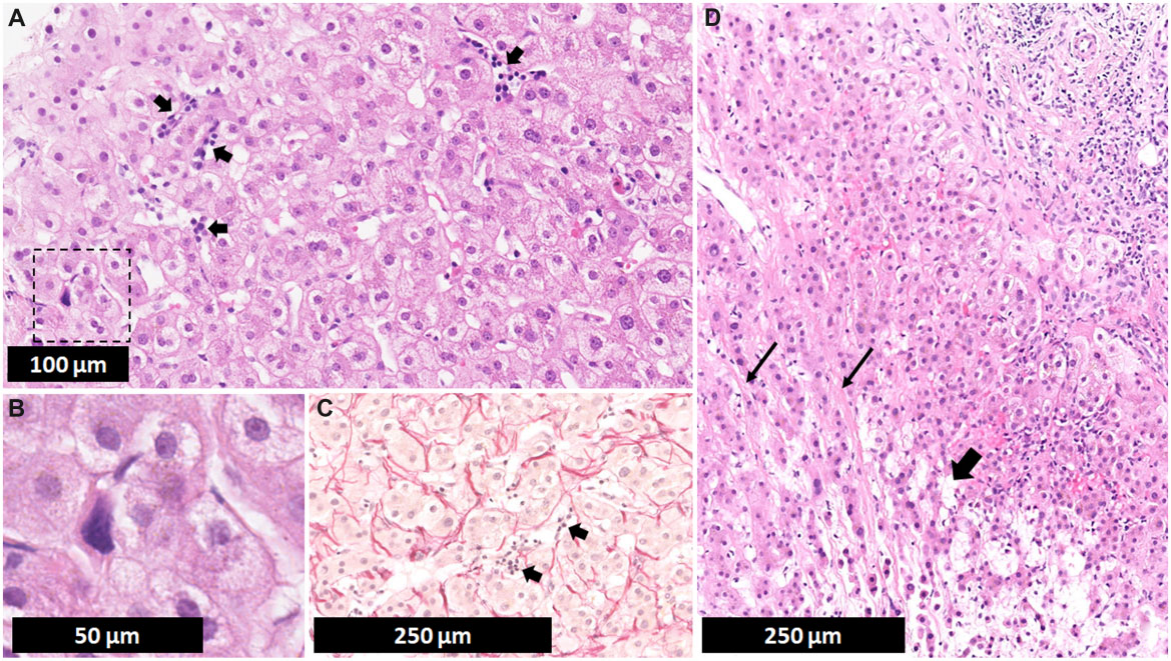
Liver needle biopsy: histopathological examination. (A) Several foci of extramedullary hematopoiesis are present (arrows) with clumps of erythroid and myeloid precursors (H&E). (B) Higher magnification of rectangle in (A). Isolated megakaryocytes are also observed. (C) A sirius red stain (collagen fibers in red) shows prominent perisinusoidal fibrosis in the vicinity of clumps of extramedullary hematopoiesis (arrows). (D) Foci of sinusoidal dilatation (broad arrow) and prominent perisinusoidal fibrosis (fine arrows) (H&E).

The patient underwent further diagnostic work-up. Transjugular liver biopsy and hepatic venous pressure gradient (HVPG) measurement were performed, confirming the presence of portal hypertension (HVPG of 12 mmHg). Histopathological examination showed no signs of rejection. Sinusoidal dilatation was observed, with prominent perisinusoidal fibrosis, but only mild periportal fibrosis without fibrous septa formation. Extramedullary hematopoiesis was observed with clumps of erythroid and myeloid precursors together with megakaryocytes in the sinusoids (Figure 1). A reticulin stain did not reveal signs of nodular regenerative hyperplasia (NRH). Diagnosis of NRH on needle biopsy is however difficult.

A bone marrow biopsy showed post-ET myelofibrosis (PET-MF).

In this patient, the diagnosis of post-ET with extramedullary hematopoiesis in the post-transplant liver was made. This caused portal hypertension and ascites formation. Given the symptomatic character of the disease, treatment with ruxolitinib, a non-selective JAK1/JAK2 inhibitor, was started. After multidisciplinary consultation, transjugular intrahepatic portosystemic shunt (TIPS) placement was chosen as the treatment of choice for the patient’s ascites, rather than diuretics because of his CKD and dialysis dependence. The post-operative period was favorable with the disappearance of ascites within 1 month.

## Discussion

Myelofibrosis, a clonal stem cell disorder, is one of the chronic Philadelphia chromosome-negative myeloproliferative diseases [1, 2]. Myelofibrosis is characterized by cytopenia, unexplained fibrosis in the bone marrow, and clonal proliferation of myeloid cells, leading to extramedullary hematopoiesis, hepatosplenomegaly, and constitutional symptoms (e.g. fatigue, night sweats, weight loss, and fever) [2, 3]. Myelofibrosis is the most aggressive disorder of the myeloproliferative diseases and may present as primary myelofibrosis (e.g. *de novo*) or evolve from ET (PET-MF) or polycythemia vera (PPV-MF) [2]. In our case, diagnosis was made through the bone marrow biopsy, showing PET-MF. Sinusoidal dilatation and perisinusoidal fibrosis in a liver biopsy can be seen in a wide variety of diseases and is reported in 50% of patients with myelofibrosis; it is thought to be due to obstruction by the extramedullary hematopoiesis [4].

Abdominal manifestations have been recognized as important features of this disorder. Hepatomegaly and splenomegaly are common, as opposed to ascites, which are rare and usually develop in the context of a well-established disease. The formation of ascites in myelofibrosis is often attributed to portal hypertension [1, 3].

According to the ESMO guidelines, the treatment of PET-MF depends on the international prognostic scoring system (IPSS) and Dynamic IPSS (DIPSS) [5, 6]. Allogeneic stem cell transplantation is the only curative option for these patients, but the transplant-related mortality is high [5, 7]. Hence, the treatment is essentially palliative and is generally guided by the predominant symptoms, such as anemia and splenomegaly. The standard of care for patients with low risk or intermediate-1 risk who are asymptomatic is observation. Ruxolitinib is the treatment of choice in symptomatic patients. For patients with intermediate-2 and high risk, allogeneic stem cell transplantation is the first-line treatment. If the patient is not eligible, ruxolitinib can be given or the patient can be included in a clinical trial [5].

Ruxolitinib is an oral JAK1/JAK2 inhibitor. Dysregulated JAK-STAT signaling is present in all myelofibrosis patients [5]. Ruxolitinib is not selective for the mutated *JAK2* and can therefore be used in both *JAK2*-mutated and *JAK2*-unmutated myelofibrosis [5, 8]. It induces a significant spleen volume reduction, an improvement in constitutional symptoms, as well as a survival advantage on long-term follow-up [8]. In our case, the patient was classified in the IPSS lower-risk group (low/intermediate-1). Given the symptomatic character of the disease, ruxolitinib was started in combination with an erythropoiesis-stimulating agent for the renal anemia component and acyclovir to prevent herpes reactivation.

Symptomatic treatment of the ascites should be started. The treatment of choice is diuretics. Ascites refractory to diuretics should be treated with paracentesis with albumin infusion. An equivalent option is the placement of TIPS, especially in patients with preserved liver function [1]. Since our patient had already received chronic hemodialysis, TIPS placement was performed.

## Conclusions

The formation of ascites may be the first manifestation of a myeloproliferative disorder. Extramedullary hematopoiesis should be included in the differential diagnosis of ascites. This is the first case report of TIPS placement for extramedullary hematopoiesis in a liver transplant patient. Our patient responded well to TIPS but the long-term prognosis will depend on the behavior of the PET-MF.

## Authors’ Contributions

Literature research and writing manuscript: L.D. Supervision writing manuscript, treating physician, and hepatology expert: X.V., H.D., H.V., A.G. Supervision writing manuscript and pathology expert: A.H.
